# Modeling root system growth around obstacles

**DOI:** 10.1038/s41598-020-72557-8

**Published:** 2020-09-28

**Authors:** Wencheng Jin, Jayde Aufrecht, Fernando Patino-Ramirez, Heidy Cabral, Chloé Arson, Scott T. Retterer

**Affiliations:** 1 Energy and Environmental Science and Technology Directorate, Idaho National Laboratory, Idaho Falls, 83415 USA; 2 Earth and Biological Sciences Directorate, Pacific Northwest National Laboratory, Richland, 99352 USA; 3 School of Civil and Environmental Engineering, Georgia Institute of Technology, Atlanta, 30332 USA; 4 Energy and Environmental Sciences Directorate, Oak Ridge National Laboratory, Oak Ridge, 37830 USA

**Keywords:** Computational models, Plant morphogenesis

## Abstract

State-of-the-Art models of Root System Architecture (RSA) do not allow simulating root growth around rigid obstacles. Yet, the presence of obstacles can be highly disruptive to the root system. We grew wheat seedlings in sealed petri dishes without obstacle and in custom 3D-printed rhizoboxes containing obstacles. Time-lapse photography was used to reconstruct the wheat root morphology network. We used the reconstructed wheat root network without obstacle to calibrate an RSA model implemented in the R-SWMS software. The root network with obstacles allowed calibrating the parameters of a new function that models the influence of rigid obstacles on wheat root growth. Experimental results show that the presence of a rigid obstacle does not affect the growth rate of the wheat root axes, but that it does influence the root trajectory after the main axis has passed the obstacle. The growth recovery time, i.e. the time for the main root axis to recover its geotropism-driven growth, is proportional to the time during which the main axis grows along the obstacle. Qualitative and quantitative comparisons between experimental and numerical results show that the proposed model successfully simulates wheat RSA growth around obstacles. Our results suggest that wheat roots follow patterns that could inspire the design of adaptive engineering flow networks.

## Introduction

Root system architecture (RSA) is a signature of plant development: root growth controls plant development and vice versa. Plants deploy roots to forage heterogeneous and dynamic soil environments and extract resources^1^. Roots transport nutrients for photosynthesis and growth, while also providing anchorage for the plant^2^. In the last decade, the dynamic interactions between the RSA and the soil matrix have received a lot of attention^3^. Investigating root growth in situ is challenging because plant growth takes time and observation through opaque soil requires expensive equipment (e.g., a micro-computed topographer). Importantly, the RSA presents non-negligible variability even for the same species and environmental conditions. Numerical models are thus valuable tools to systematically investigate the influence of various environmental factors on the growth of the RSA and on the productivity of whole plant^4–6^.

Numerical models of root growth in a soil matrix can be grouped into two categories: (1) continuum models, in which field variables, such as root length density and root apical meristems are defined by means of probability density functions that are updated through diffusion differential equations, to reflect root growth^7–10^; (2) discrete models, in which the components of the RSA (e.g., the order of roots, the branching points, the spatial location of each root segment) are explicitly described, and in which the growth of RSA depends both on the soil environment (e.g., pore pressure, nutrient concentration, temperature) and on predefined input variables that are specific to the genetics of the species under study (e.g., elongation rate, branching space and branching angle)^11–18^. Different discrete models were proposed for different objectives. For example, RootTyp^16^ allows simulating specific growth patterns for different plant species; SimRoot^17^ requires fewer assumptions on the growth pattern and assigns a time-dependent probability density function to each input parameter; RootMap^11,12^ includes a variety of functions to account for the diverse interactions between the root and the soil environment; SPACSYS^18^ focuses on the distribution of photosynthate from the shoot; R-SWMS^14,15^ is the state-of-art code that accounts for solute and water flow in soil and root system; the RootBox^13^ employs a novel L-system to mathematically describe the RSA under the assumption that the morphology of the RSA is self-similar (e.g., fractal); and the DigR^19^ features RSA analysis and root type classification. Detailed comparisons of all the discrete models in the literature are provided by Dunbabin et al.^20^ and Warren et al.^21^. State-of-the-art discrete models allow simulation of root growth in response to different soil environments. In particular, these models allow one to account for the spatial and temporal variability in water contents, nutrient concentration, pesticide concentration, and macro pore distribution^22^, as well as for the presence of bacteria^3^. However, no satisfactory models exist to date for modeling root growth around rigid obstacles. Yet, the presence of obstacles can be highly disruptive to the root system, and it is important to understand how plants can develop a resilient system of roots that ensure water and nutrient uptake, even in harsh environments.

Wilson^23^ was the first to study root growth around obstacles when discovering that maple tree roots curve back to their original growth direction after passing an obstacle. An in-depth analysis later revealed that six genes are involved in the relation between the obstacle contact stimulus and the root growth direction response for arabidopsis^24^. Specifically, the contact stimulation at the root cap regulates geotropism so that roots grow along the obstacle boundaries^25^. Based on the observation of 4 grass species, it was found that the stimulus-response relation is enabled by the accumulation of root exudates at the root cap^26^. In addition to changing root morphology, studies also showed that obstacles affect the elongation rate, the total root length, as well as the withering extent of the lateral roots relative to the major root in contact with the obstacle^26,27^. To the authors’ best knowledge, of all continuum and discrete models proposed in the literature, only the model developed by Dunbabin et al.^28^ can simulate root growth around obstacles in the code RootMap. However, this function does not allow simulating the growth of roots passed the obstacles, and it was never validated against any in situ or laboratory experiments. Note that other discrete models have the capability to consider the interactions of roots with obstacles. For example, DigR^19^ explicitly provides a plugin feature to incorporate any environmental influence on root growth behavior. The challenging part is the quantification of the behavior and its implementation in specific codes. Another challenging part for discrete models is parameter calibration, because differences in the parameterization of RSA traits lead to significant changes in the modeling results^1,29^. The latest techniques employed to mitigate this challenge, including X-ray computed tomography^30^, magnetic resonance imaging^31^, and neutron radiography^32^, still require elaborate data processing and are only suitable for relatively simple root systems. Additionally, these techniques lose significant accuracy when roots are in contact with an obstacle or chamber boundaries. It is argued that parameterization through 2D images is a good alternative since images can be either automatically processed with generic software^33,34^ or manually handled with high accuracy.

In this paper, an enrichment function is proposed to simulate root growth around obstacles, based on the observation of 2D wheat growth experiments on agar. The function is implemented in R-SWMS and calibrated against experimental results. We first present the governing equations that are implemented in R-SWMS and the experimental methods to grow wheat on petri dishes and in rhizoboxes (“Theory and methods”). The experimental results without obstacle on petri dishes are extracted and analyzed to supply input parameters of the R-SWMS model, and we parametrize the morphology of the root network in rhizoboxes with an obstacle, which allows modeling the RSA dynamics around obstacles (“Experimental results”). Then, we explain how we calibrated the rest of the input parameters of the R-SWMS model through sensitivity analyses (“Numerical modeling and discussion”), and a new root growth function is implemented in R-SWMS and calibrated against the experimental results. A discussion is presented in the “Conclusion” section. Due to the limited scope of our experiments, conclusions cannot be generalized to all plants, but our simulation results can provide inspiration for designing adaptive engineering networks.

## Theory and methods

### The R-SWMS model

To simulate Root System Architecture (RSA) evolution numerically, we use the R-SWMS model, initially developed by Somma et al.^35,36^ and enhanced by^15^ and^37^. The R-SWMS model couples root growth with fluid flow and solute transport in both soil and roots using the Finite Element Method (FEM). In the present study, we only consider water flow in unsaturated soil/substrate, water flow in the root xylem, and water uptake. Water transport equations stem from the classical Richard equation combined with Van Genuchten relation^38^, as explained in Table 1. The Van Genuchten equation relates soil conductivity *K* and water content $\theta$ with water pressure head *h*. The water flux in the root xylem has an axial component, calculated by Darcy’s law, and a radial component, calculated by a linear flow equation that relates the pressure difference between the soil and the root segment ($h_s-h_x$) with the radial water flux ($q_r$). Water uptake is modeled as a sink term (*S*), which appears in the water flow equations both in the soil and in the roots. All the equations listed in Table 1 are solved using the FEM, with an iterative solver originally coded by Simunek et al.^39^.

**Table 1:** Governing equations of the “R-SWMS” model.

**Soil water flow**	$\frac{\partial \theta }{\partial t} = \nabla \cdot {K} \nabla (h+z) -S$
	$$\theta (h)= \theta _r+\frac{\theta _s-\theta _r}{(1+(\alpha |h|^n))^m}$$
	$${K}(h)= K_s\{ 1- \frac{(\alpha |h|)^{n-1}}{[1+(\alpha |h|^n)]^{-m}} \}^2 [1+ (\alpha |h|^n)]^{-lm}$$
	$$m=1-1/n$$
**Xylem water flow**	$q_r = L_r(h_s-h_x), \quad J_r=2 \pi r q_r \text {d} l$
	$$J_x= -K_x\Big ( \frac{\text {d} h_x(z)}{\text {d} l_{seg}} + \frac{\text {d} z}{\text {d} l_{seg}} \Big )$$
**Water uptake**	$S= \frac{1}{|V|} \int _V \delta _2 \big ( \chi ( {\varvec{x}} ) \big ) q_r \text {d} {\varvec{ x}}$
$\theta$: soil water content	$\theta _r, \theta _s$: residual/saturated $\theta$
*K*: unsaturated conductivity	$h_s, h_x$: pressure heads at the soil-root interface and inside the xylem
*h*: water pressure head	$K_s$: saturated hydraulic conductivity
*S*: source/sink term	*l*: pore connectivity parameter
*z*: depth	$q_r$: radial flux density
$L_r$: radial conductivity	$J_r$: radial volumetric flow
$J_x$: axial volumetric flow	$l_{seg}$: root segment length
$K_x$: xylem axial conductance	$\alpha , n$: water retention curve shape parameters
$\delta _2()$: 2D Dirac delta function	$\chi ({\varvec{x}})$: characteristic function^40^

**Figure 1:**
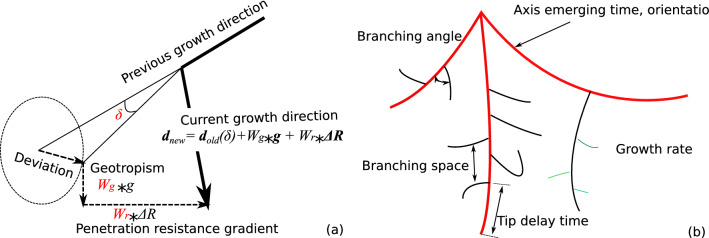
(**a**) Method to determine root growth direction in the R-SWMS model; (**b**) sketch of root growth morphological traits. Note that axis, second-order and third-order roots are color-coded by red, black, and green lines. Axis emerging time and orientation refer to the time at which each axis starts to germinate and to its initial growth direction, respectively. Branching angle is the average angle between axis and secondary roots, between second-order and third-order roots. The branching space is the average distance between the two nearby branches. The tip delay time is the time elapsed from root germination to its first branch germination. The growth rate is the elongation rate of for the root order considered.

The geometrical characteristics of the RSA are defined, updated and stored in vectorized variables at every predefined and fixed time step. Root growth at each tip is calculated based upon the plant’s genetic characteristics (input parameters) and upon the values of the environmental conditions such as soil compressibility, water pressure head, and gravity forces in the finite elements that contain the root tips. Field variables such as water pressure head are then updated at each time step by balancing the water flow in the soil elements with the water flow in the roots, using water uptake as the sink term. Note that the time step used for field variable update is iteratively adjusted based on convergence rate, and in general, this time step is smaller comparing to the time step for RSA update. Multiple increments of FEM calculation for field variables are needed to match with the RSA update time increment. The gene-specific evolution law of the plant dictates the root growth rate, the number of main axes, the branching angle, the secondary root spacing, the order of appearance of the secondary roots—to name only a few. The local soil environment affects both the direction of root growth and the root elongation rate. A soil penetration resistance *R* (kPa) is defined to quantify this influence, as follows:

1 $$\begin{aligned} log_eR = 0.35 \times log_{10}(|\psi | \times S_e) + 0.93\times \rho _b +1.26 \end{aligned}$$

where $\psi$ (kPa) is the water potential, $S_e$ is the effective saturation degree, and $\rho _b$ (g/cm$^3$) is the soil bulk density. Eq. (1) was obtained by fitting the R-SWMS model against experimental data from 12 soils with different bulk densities, organic carbon contents, sand, silt and clay contents from different depths^41^. The R-SWMS model accounts for the hydraulic interaction between the root system and the soil environment by making the growth direction and the growth rate depend on the value of water pressure and soil density, and by calculating the water uptake from the field variables in the soil environment. Figure 1a illustrates how the code calculates the root growth direction (${\varvec{d}}_{new}$) from the growth direction of the root segment at the previous time step (${\varvec{d}}_{old}$), gravity (${\varvec{g}}$) and the gradient of the penetration resistance ($\Delta R$). The root growth rate *E* is calculated by using the following expression:

2 $$\begin{aligned} E = E_{max} \times \left( 1 - \frac{R_{ave}}{4000+2.33\times |\psi |}\right) , \end{aligned}$$

where $E_{max}$ is the maximum elongation rate, measured directly from experiments, and $R_{ave}$ is the average penetration resistance at the growing root tip.

### Wheat root growth experiments without obstacle

In order to obtain root growth morphological traits (Fig. 1b), root growth experiments were conducted. Winter wheat (*Triticum aestivum*, Prairie Moon Nursery) was chosen as a model organism for this study because of its rapid growth, compatibility with agar-based growth substrates, and economic significance. The seeds were stored at $4\,^\circ$C until the beginning of the experiment to simulate winter and encourage germination. At the start of each experiment, stratified seeds were surface sterilized by rinsing for 5 minutes with an aqueous solution of 30% bleach and 0.1% TritonX and then rinsed 4 times with sterile water. Sterilized seeds were then transferred to a petri dish filled with 1/4$\times$ Murashige–Skoog media and 7% agar. The petri dishes were sealed with Parafilm and placed vertically in a 12 h light/dark cycling growth chamber at $23\,^\circ$C overnight. The following day, germinated seedlings free of contamination were chosen for the experiments.

**Figure 2:**
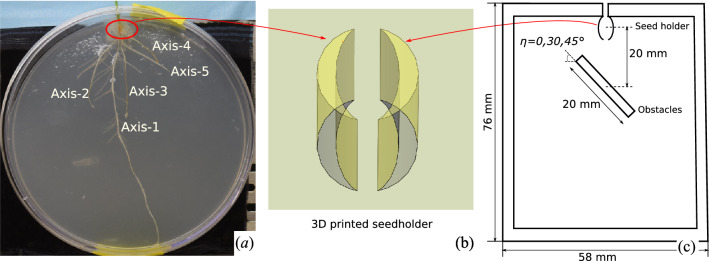
(**a**) Time-lapse photographic image of one of the wheat root growth experiments; (**b**) sketch of the 3D printed seed holder; (**c**) sketch of the 3D printed growth chamber with obstacles. Note that the chamber was sealed during the experiment except for the top hole, which allowed the shoot to grow out of the chamber. The growth domain (inner rectangle) had a surface area of $48\times 68$ mm$^2$.

In order to support the seedling during growth and eliminate bias in seeding orientation, “seed holder” (Fig. 2b) was designed and 3D printed using a stereolithography printer (Form Labs 2) and attached to the bottom of a 100 mm diameter Petri dish (VWR) using a polyurethane glue (Duco Cement). The petri dish was modified further to permit the emerging wheat shoot to grow upwards by drilling a 5 mm hole in the side of the dish with a Dremel drill. Once modified, the dish was sterilized for 15 min in a UV stratalinker oven.

The modified petri dish was filled with a 40 mL aqueous solution of 1$\times$ Murashige–Skoog (4.3 g/L) plant media and 9% agar. Working in a biosafety hood, the germinated seedling was transferred to the seed holder of the dish with sterile forceps, and the emerging shoot was threaded through the 5 mm opening. The dish was sealed using Parafilm (the 5 mm opening unsealed) and oriented vertically to encourage root geotropism. The biosafety hood provided constant light to the seedling throughout the week-long experiment and the sealed dish provided adequate moisture and nutrients. A Nikon DSLR camera mounted on a stand was used in combination with an automatic shutter timer remote (Vello Shutterboss II) to image the morphology of the wheat root every 12 h for a week. Figure 2a shows the obtained root image.

### Wheat root growth experiments with rigid obstacles

In order to quantify the response of roots to rigid obstacles, we carried out another series of experiments to investigate the variation of growth rate and growth direction of wheat roots before, during, and after they contact with rectangular obstacles. For these experiments, custom rhizoboxes were designed and 3D printed using a stereolithography printer (Form Labs 2) (Fig. 2c). The outer dimensions of the rhizoboxes were 7.6 cm $\times 5.8$ cm and each rhizobox was designed with a seed holder and a 5 mm opening for the emerging wheat shoots (see “Wheat root growth experiments without obstacle” section). In these experiments, the seed holder ensured that the roots were emerging exactly 20 mm above the center of the obstacle. The obstacles were all 20 mm in length and varied in orientation with respect to the bottom of the seed holder $(\eta = 0, 30, 45 ^\circ )$. The rhizoboxes were also designed with slots to fit a 64 mm $\times$ 50 mm glass slide so that the rhizobox could be covered once the seed was placed in the holder.

To provide water and nutrients to the growing seedling, the 3D printed rhizoboxes were filled with 1$\times$ Murashige–Skoog media and 9% agar until the solution was flushed with the bottom of the seed holder. Once the sterilized seedling had germinated, it was transferred to the seed holder using sterile forceps in a biosafety hood. A glass microscope slide was then fitted into the rhizobox on top of the seed to permit imaging and minimize drying of the agar. The seedlings were oriented vertically and kept in a biosafety hood which prevented contamination and provided constant light to the seedlings. Using the same imaging set up as that described in Subsection “Wheat root growth experiments without obstacle” section, photographs of the roots were taken every 2 h. Three replicate experiments were conducted for each obstacle orientation angle $(\eta = 0, 30, 45 ^\circ )$. Figure S1 shows the root systems of wheat growing around obstacles within the rhizoboxes at different growth times (*t*).

## Experimental results

### Root growth morphological traits

Extracting root growth information from time-sequential images is not trivial. Many image-analysis algorithms have been proposed in the literature^34,42,43^. However, due to the presence of roots crossing each other in our experiments, we could not handle the process automatically. We imported each time-lapse photograph into AutoCAD and we meticulously drew the lines forming the root architecture manually. In the following, an axis is defined as a root segment that emerges from the seed (from seed to tip), and a branch is a root segment that emerges from a branching point. From the AutoCad drawing, we calculated the total length of each root segment, including the axes. The growth rate for each of the root axes and branches was calculated from the lengths measured over known growth periods.

**Figure 3:**
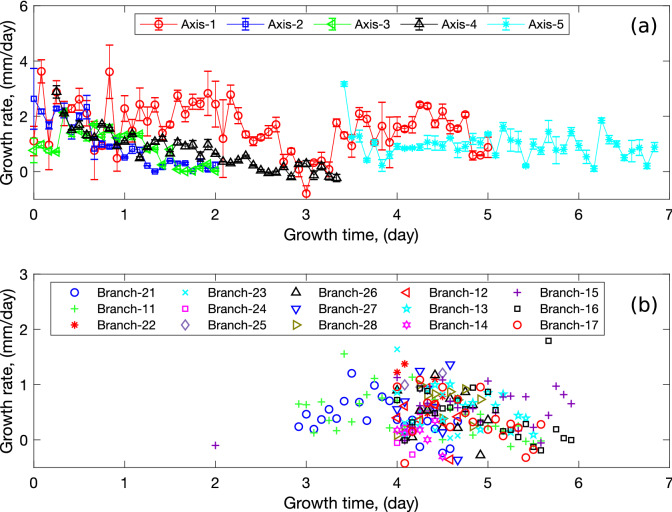
Growth rate evolution of (**a**) first order root segments (axes) and (**b**) second order root segments (lateral roots) with growth time extracted from wheat growth laboratory experiments. The error bar represents the standard deviation of growth rate from three measurements. Note that the axes and lateral branches are named after their emerging time: branch-21 is the the first emerging branch from axis-2. The negative value is due to human error from AutoCAD measurement.

**Figure 4:**
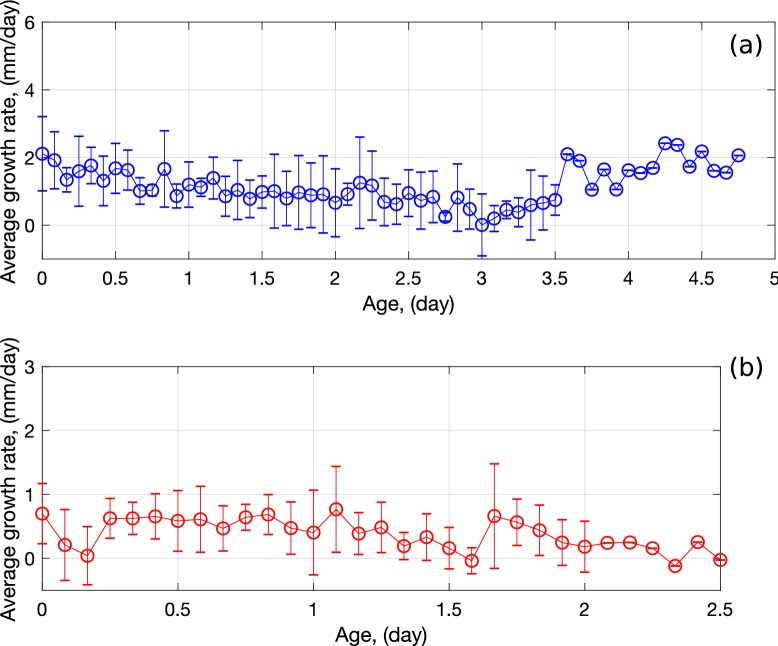
Average growth rate evolution of (**a**) first order root segments (axes) and (**b**) second order root segments (lateral roots) with age extracted from wheat growth laboratory experiments. The error bar represents standard deviation of growth rate from all measurements. In average, growth rate of axes decreases with aging, while growth rate of secondary roots keeps constant.

The growth rate for each axis is presented in Fig. 3a, in which the growth time starts from the time of seed germination. Note that axes were named after their emerging date (e.g., axis 1 appeared first, axis 2 appeared second—see Fig. 2). The average growth rate of the axes was calculated and then expressed as a function of axis age for future input in the R-SWMS model (Fig. 4a). In the experiments studied here, the average axis growth rate decreases during the first 3.5 days. Beyond that period of time, only the main axis (axis 1) grows—which explains the absence of an error bar in Fig. 4a. Figure 3b shows the growth rates of the second-order branches, called lateral roots, which were calculated by following a similar procedure. Different from the axis root growth rate, the average lateral root growth rate stays almost constant throughout the 2-day period when they are observed (Fig. 4b). Lastly, the average branching angle was found to be $32.55^\circ$ and the average branch spacing was 0.3 cm.

### Root growth around rigid obstacles

Following the same procedures as in the “Root growth morphological traits” section, we imported the photographs taken during the experiments (Figure S1) into AutoCAD, and we manually measured the length of each root axis. At all times, the growth rate was calculated by using the length difference between two images taken at a fixed interval of 2 h. Figure S2 shows the extracted growth rate for obstacles inclined at an angle $\eta = 0^\circ ,\; 30 ^\circ ,\; 45^\circ$. The results do not show any significant variation after the major axes touches the obstacle (time zero with the shifted time axis). The growth rate increases by the same amount in a synchronic manner, which indicates that the nutrients are equally distributed among axes. We conclude that the growth rate is not significantly influenced by the contact of the major axis with a rigid obstacle, and this observation holds for all the obstacle orientations tested in the experiments.

**Figure 5:**
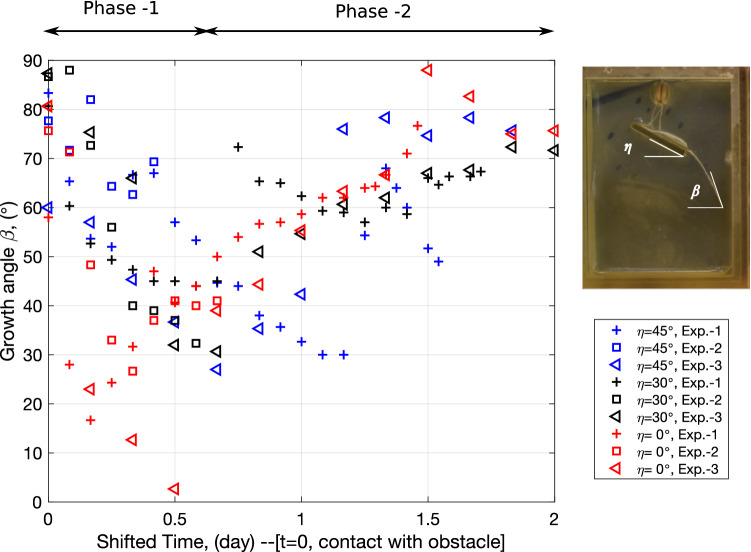
Extracted growth angle $\beta$ of the root axes after the major axis has hit the obstacle, for different obstacle orientation angles $\eta$.

The evolution of the growth direction after the major axis has hit the obstacle is shown in Fig. 5. The tip growth angle of the major axis, noted $\beta$, is measured in AutoCad. Due to the exploratory nature of the root growth process, not all major axes start growing vertically, i.e., with a growth angle $\beta =90^\circ$. After entering in contact with the obstacle, the major axis aligns itself with the long edge of the rectangular obstacle, which translates into a monotonic decrease of the growth angle $\beta$, which ends up being equal to the obstacle inclination angle $\eta$. The period during which the major axis grows along the obstacles is called “Phase-1”. In the subsequent phase, called “Phase-2”, the growth angle gradually increases to go back to its original value, close to $90^\circ$. Note that the partition of time into “Phase-1” and “Phase-2” is an approximation, since it is averaged over all the replicates. A detailed analysis of the images drawn in AutoCad indicates that the period of time of Phase-2 is proportional to that of Phase-1. Physically, this means that the time necessary for the root to recover its trajectory increases in accordance with the time during which the root was in contact with the obstacle. We note that data points are sometimes scattered among replicates of the same experiment, which indicates that other factors than the presence of the obstacle, likely genetic factors, influence the growth angle. For example, in one of the experiments with $\eta =45^\circ$, shown in Figure S1, the major axis deviates from the obstacle before it reaches the end of the obstacle.

## Numerical modeling and discussion

### Numerical calibration of R-SWMS model parameters

In addition to the root growth rate, root branching angle, and root branch spacing, which can be determined experimentally, the R-SWMS model requires four more input parameters: the tip delay time (defined as the time needed for the root to grow its tip from the closest branching point, as shown in Fig. 1a), the relative weight of geotropism on the root growth direction, the relative weight of the substrate penetration resistance gradient on the root growth direction and the maximum random deviation angle (used to mimic the dynamic exploration of the root tip). To calibrate these parameters, the influence of which is illustrated in Fig. 1b, we first simulate wheat root growth with the average axis growth rate, the secondary branch growth rate, the average branching angle, and the average branch spacing measured experimentally, for various values of tip delay time, geotropism weight, substrate penetration resistance weight, and maximum random deviation angle. It is not practical to calibrate the RSA model by expecting a pixel-to-pixel match between experimental and numerical results, due to the stochastic variability of root systems in nature. So instead of checking the position of every point of the root system, we calculate several network indices (defined below) and we compare the network indices obtained numerically to those determined experimentally. The least-square method is adopted to find the best fit.

**Figure 6:**
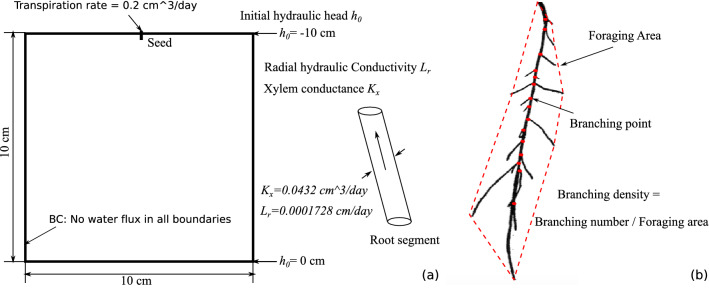
(**a**) FEM initial and boundary conditions used for the calibration of the R-SWMS model parameters; (**b**) definition of the network indices for numerical calibration.

The initial and boundary conditions adopted for the FEM simulations are similar to those of the physical experiments, as illustrated in Fig. 6a. A pseudo-2D model of size $10\times 10\,\mathrm{cm}^2$ with a thickness of 0.2 cm is used to represent the growth domain of the petri dish. All the boundaries of the domain are undrained (no water flux). Initially, the water table is at the bottom of the domain, i.e $h_0=0$ cm is set as an initial boundary condition. The parameters used to characterize fluid flow in the substrate and along the xylem are listed in Table 2. Note that the xylem radial conductivity and axial conductance are expressed per unit length of the root segment. We used a 0.2 cm$^3$/day constant flow rate at the root collar, which corresponds to the typical range of transpiration rate for wheat. Note that the substrate penetration resistance is calculated in terms of water potential, effective saturation degree, and substrate bulk density^41^. For all the calibration simulations presented in this section, we used a substrate density of $\rho _{b} = 1.2 \,\mathrm{g/cm}^3$ (typical for soils).

**Table 2:** Fluid flow parameters used for the calibration of the R-SWMS model parameters.

$$\theta _r$$	$$\theta _s$$	*a* (cm$$^{-1})$$	*n*	$$K_s$$ (cm/day)	$$K_x$$ (cm$$^3$$/day)	$$L_r$$ (cm/day)
0.06	0.41	0.03	2.5	10.24	$4.32\times 10^{-2}$	$1.73\times 10^{-4}$

Figure 6b illustrates the three network indices used in this study: the total length of the root network, the foraging area, and the branching density. Note that no lateral root was noted around axes 3, 4, or 5 in our experiments (Fig. 2a). Therefore, we used the foraging area and the branching density of axes 1 and 2 only for the calibration procedure.

The tip growth direction influences the RSA but not the total root length index. The only unknown input parameter that controls the root total length is the tip delay time, so we first calibrated the tip delay time by comparing the total length of the root system in the numerical simulations to those in the experiments (Figure S3). The experimental results show that root growth operates in two stages: (1) Development of axes (the first-order roots) during the first 3.5 days, and (2) extensive secondary lateral root growth in the following days. Numerical simulations capture the phenomenon, and the two-stage transition point is directly affected by the tip delay time parameter. The best fit in terms of total root length is obtained for a tip delay time of 2.1 days.

**Figure 7:**
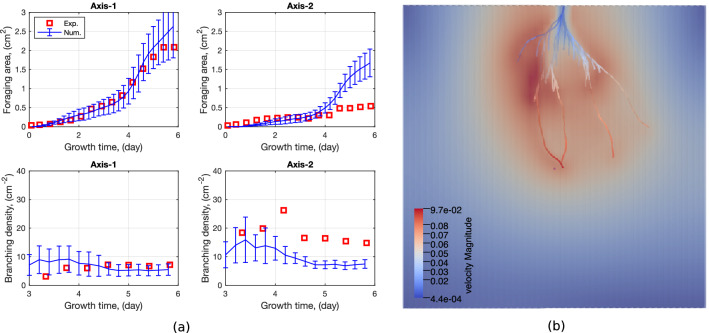
Calibration of $W_g$, $W_r$ and $\delta$, within narrow value intervals. The best match between experimental and numerical results was found by comparing the foraging area and the branch density over time, for the two root axes that develop lateral roots. Note that in the simulation results shown in b, the roots are color-coded with the value of the hydraulic head. (**a**) Calibration of RSA input parameters by network index comparison. The error bar represents standard deviation of the indices from 10 numerical simulations; (**b**) simulated root network with the calibrated wheat root growth parameters using R-SWMS^15^.

For the remaining three input parameters (geotropism weight $W_g$, substrate penetration resistance weight $W_r$ and maximum random deviation angle $\delta$), we carried out an extensive parametric study to minimize the difference between the experimental and numerical values of the foraging area and of the branching density over time. The FEM mesh, initial conditions, and boundary conditions were the same as previously (see Fig. 6a). We used the experimental values of the average axis growth rate, the secondary branch growth rate, the average branching angle, and the average branch spacing. The fluid flow parameters were the same as in the previous simulations (see Table 2). The tip delay time was taken equal to 2.1 days, according to the previous calibration. In order to narrow down the range of the three unknown input parameters, we simulated root growth with two parameters fixed and one parameter varying within a range of realistic values (i.e. $W_r = 0.4, \delta = 50^\circ , W_g \in [0, 0.5]$; $W_g = 0.2, \delta = 50^\circ , W_r \in [0, 0.5]$; and $W_g =0.2, W_r = 0.4, \delta \in [10^\circ , 50^\circ ]$). Through the comparison of these numerical results against the experimental data (Figures S4–S6), we found that the input parameters should be in the following ranges: $W_g \in [0.1, 0.2]$, $W_r \in [0.35, 0.45]$, and $\delta \in [25^\circ , 35^\circ ]$. We performed another set of simulations by varying all three input parameters simultaneously within the identified ranges, and we found that the combination of parameters $W_g = 0.1$, $W_r = 0.45$, and $\delta =35^\circ$ minimized the error between the experimental and numerical indices of foraging area and branch density over time. Figure 7a shows the comparison between the experimental and numerical network indices, in which numerical simulations were run with the calibrated parameters 10 times, and the error bars represent the standard deviation of the 10 simulation results. Figure 7b shows the root system simulated with R-SWMS after 6 days of growth, with the calibrated parameters. Note that we plotted the branching density after 3 days of growth, since the index of branching density is only meaningful when laterals are developed. Numerical results indicate an exponential increase in foraging area and a quasi constant branching density, which captures well the topological pattern of axis-1 throughout the growth period (see Fig. 2). However, the numerical prediction significantly deviates from the experimental results after 4 days of growth. This is because axis-2 stops growing after 2 days of age in the experiment (Fig. 3a). We performed a network analysis to compare quantitatively the experimental and numerical root systems. The results, summarized in the Appendix, show that the R-SWMS model is properly calibrated.

### Numerical model of root growth around rigid obstacles

Despite stochastic and genetic variability, we observe that in all the experiments:

1. The presence of the obstacle does not have any significant influence on the growth rate of any root axis, primary or secondary;
2. Roots grow along the obstacle boundary if the obstacle stands in the way of the growth path (Phase-1 in Fig. 5);
3. Roots recover their growth trajectory after a period of time that is proportional to the time during which they were in contact with the obstacle (Phase-2 in Fig. 5).

Although these conclusions may not be true for all species or at all times beyond the typical duration of our experiments, our experiments were repeatable, so we designed a model to replicate them numerically, to allow comparisons with other species. We also provide a network analysis method that can help assessing the feasibility of bio-inspired engineering networks in the future.

The original version of the R-SWMS code does not allow simulating root growth around obstacles. We thus propose a model to allow simulating Phases 1 and 2 described above. No particular function is needed to reproduce conclusion 1 numerically, whereas conclusions 2 and 3 require implementing new functions. One possibility is to model the rigid obstacles by voids in the FEM model, i.e., by non-meshed areas. The inner boundaries of the void area are then subjected to a zero-flux boundary condition. Unfortunately, the current version of the R-SWMS code only works for structured meshes, which align element boundaries with the global coordinate axes. As a result, the obstacles have to be represented with polygons that have step-wise, zig-zag boundaries instead of straight boundaries. Although this limitation does not affect the calculation of the water flow, it does affect the calculation of the penetration resistance gradient and of the water uptake since sections of root might be located outside the simulation domain, which leads to changes in root morphology. That is why this modeling option is not practical.

We choose instead to treat obstacles as domains that contain very stiff substrate, with a high bulk density and a low permeability, for which the substrate penetration resistance is very high according to Eq. (1). This high substrate penetration resistance prevents root growth in the direction normal to the obstacle boundaries. We also set the geotropism weight $W_g$ to unity, so as to ensure root growth along the obstacles. This modeling option does not require any additional implementation on the FEM calculation of field variables, it only requires to assign different material parameters to the obstacle(s) occupied area. We only need to perform minimum implementation on the RSA update part, such as storing the geometry of the obstacles and detecting the relative position of the root tip and the obstacles.

**Figure 8:**
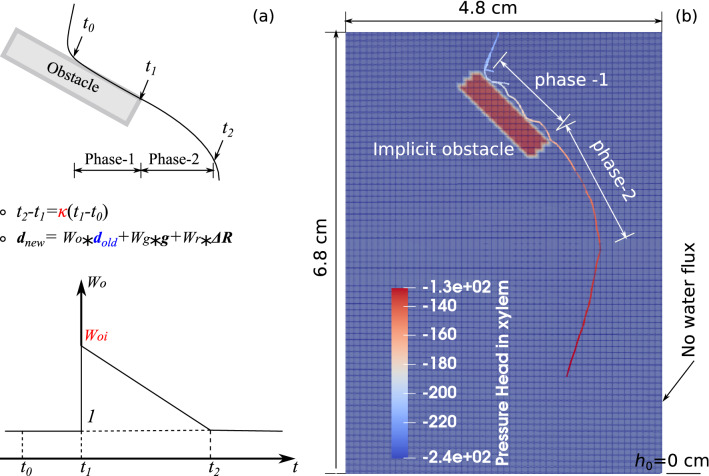
(**a**) Principle of the numerical model of root growth around rigid obstacles: the growth direction along the obstacle has a weight that has a vanishing influence over time in the calculation of the root growth direction; (**b**) simulation of root growth around an obstacle oriented at an angle $\eta =45^\circ$. The obstacle is modeled as a rigid, quasi-impermeable substrate. The growth orientation function shown in (**a**) is used. Dimensions, initial and boundary conditions are the same as in the physical experiments. The color scale indicates the distribution of the pressure head in the xylem of root system.

In the original version of R-SWMS, the growth orientation is calculated as a weighted sum of the direction of gravity $W_g{\varvec{g}}$, of the previous growth direction with a random angle ${\varvec{d}}_{old}(\delta )$, and of the direction of the substrate penetration gradient $W_r \nabla R$ shown in Fig. 1b. In order to capture the recovery effect described in conclusion 3 above, we replaced the term ${\varvec{d}}_{old}(\delta )$ with a new function $W_o {\varvec{d}}_{old}$ during Phase-2 (Fig. 8a). Figure 8a explains how the weight function $W_o$ is calculated: $W_o$ is equal to 1 between the time when the root hits the obstacle ($t_0$) and the time when the root leaves the obstacle ($t_1$), and it varies linearly between an initial weight $W_{oi}>1$ and 0, between the end of Phase-1 (at time $t_1$) and the end of Phase-2 (at time $t_2$). The time period $\Delta t = t_2 - t_1$ is called recovery time. By construction of the model, the recovery time is proportional to the time period when the root is in contact with the obstacle. We note $\kappa$ the coefficient of proportionality, such that: $\Delta t = \kappa (t_1-t_0)$. Additionally, we ignore the effect of the random deviation angle $\delta$ to better highlight the effect of the obstacle on the RSA, i.e. we take $\delta =0^\circ$. A new function is implemented to calculate the average growth direction of the root when it is in contact with the obstacle, and this average growth direction is treated as ${\varvec{d}}_{old}$ at time $t_1$. After that, ${\varvec{d}}_{old}$ represents the growth direction of the previous time increment. The determination of $(t_0, t_1)$ pair is realized by iteratively comparing the distance between each tip and the closest point at each obstacle periphery against a threshold.

Figure 8 shows an example of a root system that was obtained numerically by modeling the obstacle as a stiff substrate and by using the root growth orientation function explained in Fig. 8a, to account for the recovery time of the root after it hits the obstacle. The bulk density $\rho _{b}$ and the saturated hydraulic conductivity $K_{s}$ within the obstacle domain (in red in Fig. 8b) were taken as $\rho _{b} = 1.4\,\mathrm{g/cm}^3$ and $K_{s} = 5$ cm/day, respectively. The values of $\rho _{b}$ and $K_{s}$ for the obstacle domain reflect a stiff, quasi-impermeable material, and are chosen so as to avoid numerical convergence issues. The dimensions of the FEM model were the same as in the physical experiments, and the elements were assigned a thickness of 0.08*cm* to mimic pseudo-2D conditions. The initial and boundary conditions (including the undrained fluid flow at all boundaries, the zero pressure head at the bottom, and the constant flow rate 0.2 cm$^3$/day at root collar) and the fluid flow parameters for the roots and for the agar were the same as in the previous section (Fig. 6a). The root growth parameters were those calibrated in previous sections. For an obstacle with an inclination angle $\eta = 45^\circ$, the simulated root system after 3 days of growth (shown in Fig. 8b) is similar to the observed major axis topology shown in Figure S1. The modeled root bends to grow along the obstacle, then continues to grow along the direction of the obstacle due to the recovery effect after the root leaves the boundary of the obstacle. This effect gradually fades away, and the growth direction returns to the vertical after the recovery period.

### Calibration root growth parameters with obstacle

**Figure 9:**
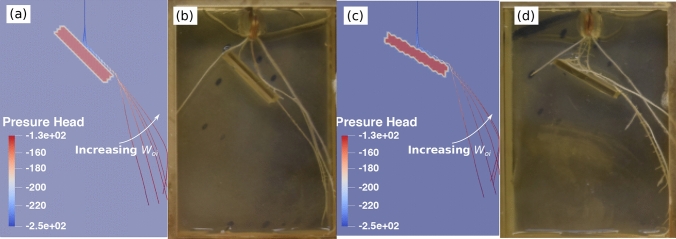
Influence of $W_{oi}$ on the RSA after the major axis hits the obstacle: comparison of numerical and experimental results. (**a**) Simulated major axis using different $W_{oi}$ with an obstacle oriented at $\eta =45^\circ$ after 3 days of growth; (**b**) physical root system obtained with an obstacle oriented at $\eta =45^\circ$ at growth time $t= 3$ days; (**c**) simulated major axis with an obstacle oriented at $\eta =30^\circ$; (**d**) physical root system obtained with an obstacle oriented at $\eta =30^\circ$ at growth time $t= 3$ days. Note that the color scale in in (**a**,**c**) marks the water pressure head along the root xylem.

The proposed model of root orientation recovery explained in Fig. 8a depends on two parameters: $\kappa$ and $W_{oi}$. From the extracted results in Fig. 5, it is found that $\kappa$ is equal to about 2. We perform a parametric study to calibrate the value of $W_{oi}$ against the experimental results. We repeat the simulation shown in Fig. 8 with $\kappa =2$ and with $W_{oi}=1,\, 2.5,\, 5,\, 7.5,\, 10$, for each of the obstacle orientations tested physically ($\eta =0^\circ ,\, 30^\circ ,\, 45^\circ$). As shown in Fig. 9, the recovery time (Phase-2) is longer when $W_{oi}$ is larger for $\eta =30^\circ$ and $\eta =45^\circ$. Additionally, it is clear that the modeled major axis does not resemble the physical one when the historical influence is not considered ($W_{oi}=1$).

**Figure 10:**
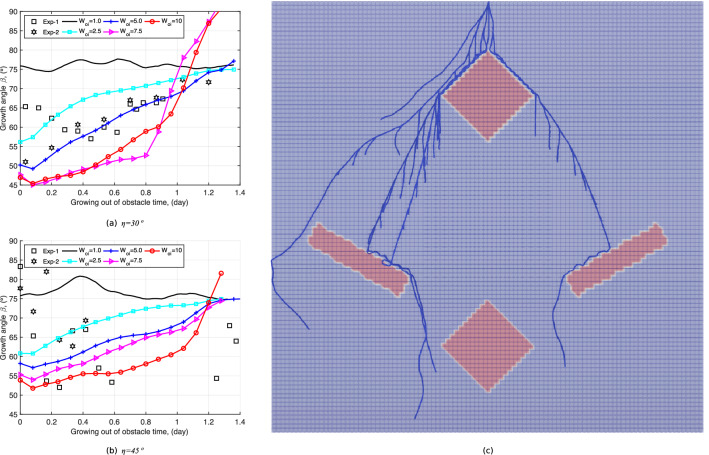
Sensitivity analysis to determine the value of $W_{oi}$ for (**a**) $\eta = 45 ^\circ$, (**b**) $\eta = 30^\circ$ by comparing the root growth angle $\beta$ (defined in Fig. 5) obtained numerically to the one obtained experimentally, after the root axis passes the obstacle; (**c**) root system growing in a domain with multiple obstacles with the parameters calibrated in the previous sections.

In order to obtain the best fit for $W_{oi}$, we extracted the growth direction $\beta$ (defined in Fig. 5) for all simulation results. To avoid statistical variation, three simulations were run for each $W_{oi}$ and each obstacle inclination angle $\eta$, and the mean value of $\beta$ after $t_1$ (defined in Fig. 8a) was plotted against the value of $\beta$ obtained experimentally during Phase-2 in Fig. 10a,b. The modeling results for $W_{oi}=1$ are clearly distinct from the rest of the simulations, and the modeled growth angle increases with time for all cases with $W_{oi} \ne 1$. The simulated growth angle best matches the experimental one for $W_{oi} =5$ when $\eta =30^\circ$ and $\eta =45^\circ$. Note that the abrupt change in the evolution of the growth angle for $W_{oi} =10$ when $\eta =45^\circ$ and for $W_{oi} =7.5, 10$ when $\eta =30^\circ$ is because the modeled major axis gets in contact with the domain boundary.

With the calibrated root growth morphological parameters found in previous Sections and the parameters of the root growth orientation function for obstacles ($\kappa =2$ and $W_{oi}=5$), we simulated a root system growing for 6 days in a domain that contains multiple obstacles. Figure 10c shows the simulation result. All the axes and the secondary roots first grow along the periphery of obstacles, as expected. The history effect on the growth direction after the axes and secondary roots get in contact with obstacles is properly captured, which shows that the proposed modeling strategy in R-SWMS is effective.

## Conclusion

In order to understand and predict root growth around obstacles, we implemented a new function in the R-SWMS code, which iteratively updates the geometry of the Root System Architecture (RSA) over time as a function of genetic parameters and environmental variables. The latter include the water pore pressure and nutrient concentration of the substrate, which are calculated with a Finite Element Method.

We first calibrated the parameters of the R-SWMS model against 2D wheat root growth experiments in the absence of obstacles. The network geometry was used to calculate the growth rate, branching angle, and branch spacing of the RSA over time. These parameters were used as inputs in the R-SWMS model. Sensitivity analyses then allowed calibrating the branching delay time, the random deviation growth angle, as well as the weights of geotropism and of the substrate penetration resistance. The RSAs obtained experimentally and numerically were transformed into undirected graphs in order to calculate indices of node connectivity and node betweenness. For comparison, indices were also calculated for fan trees and Steiner trees spanning from the same origin (seed) to the same end points (root tips) as the numerical RSA. Analyses of the network indices validated the calibration of the R-SWMS model against 2D wheat root growth experiments.

We then grew wheat roots in 3D printed chambers that contained an obstacle oriented at an angle of 0$^\circ$, 30$^\circ$, or 45$^\circ$ to the horizontal, and found that the presence of the obstacle did not affect the growth rate of the root axes, but did influence the root trajectory after the main axis had passed the obstacle. The time for the main axis to recover its geotropism-driven growth direction, which we called recovery time, was found to be proportional to the time that the main axis grows along the obstacle. We implemented a new function in R-SWMS to model the effect of rigid obstacles on root growth. The weight of that new function vanishes progressively during the recovery time. We calibrated the parameters of the new function against the experimental results. In the simulations, the obstacles were modeled as domains containing rigid and quasi-impermeable substrate. A proof-of-concept simulation with multiple obstacles showed that the implementation of the new function in R-SWMS was successful.

We conducted all of our experiments in 2D, in agar, to make it possible to visualize the RSA growing over time. In future work, we plan to use non-invasive imaging technologies on 3D substrate samples, e.g. X-ray computed tomography. Detailed 3D RSA data will allow testing the model proposed in the present study and understanding how roots and actual soil interact. Our study was limited to the first few days of growth of one species. More test cases are needed to generalize the conclusions drawn in this paper. However, our experiments were repeatable and our results suggest that wheat roots follow a pattern to accommodate the presence of obstacles. This behavior could be used to design plant-inspired infrastructure networks. Leaf venations already proved to be more effective than engineering networks in 2D^44^. Simulations with root-inspired networks could allow the design of 3D networks in complex topographical environments, with applications in underground utilities or transportation.

### Supplementary information

Supplementary material 1

## Figures and Tables

**Figure 11:**
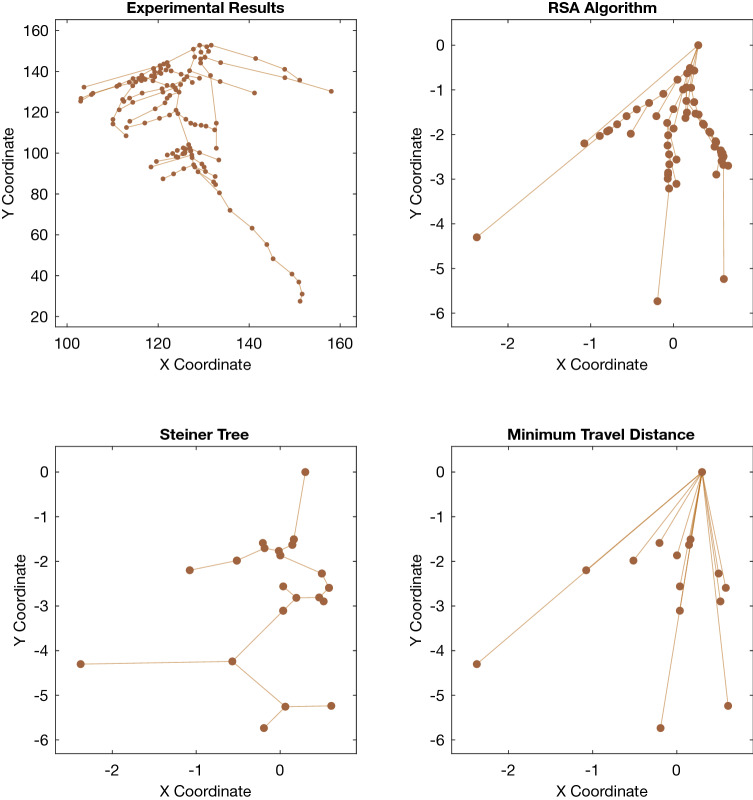
Undirected graph representations of the RSA. Top left: graph obtained from experimental results. Top right: sample results from one of the numerical simulations using the calibrated model parameters. Bottom left: Steiner tree for the same set of points as the numerical model. Bottom right: fan tree for the same set of points as the numerical model.

**Figure 12:**
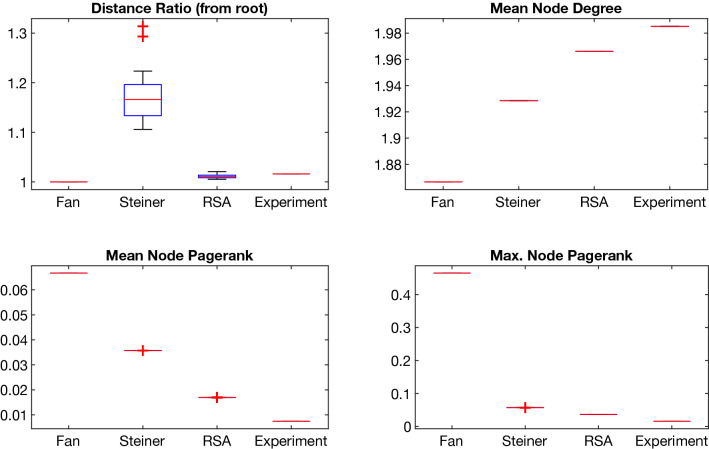
Boxplots illustrating the variability of the four different connectivity indices calculated in the undirected graphs. The red lines indicate mean values, the blue boxes provide the bounds between the first and third quartiles of the data and the red crosses indicate outliers in the distribution. The connectivity indices are independent of the size scale of the networks.

**Figure 13:**
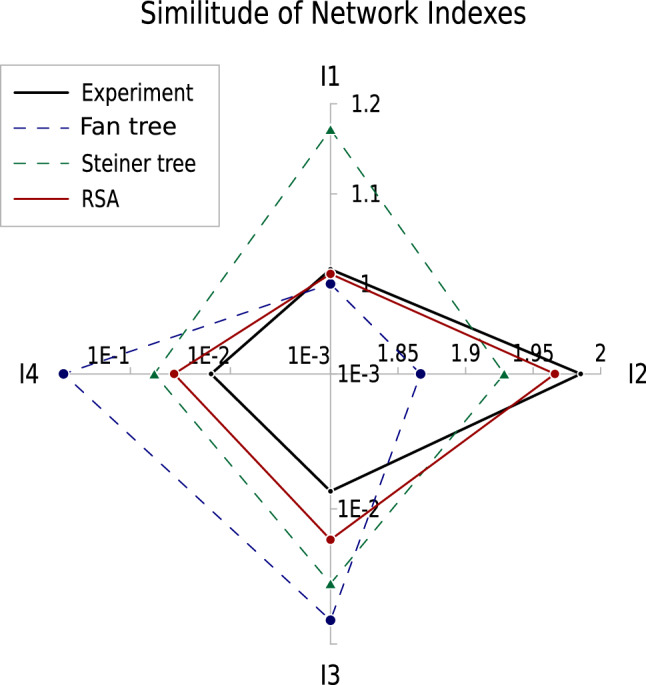
Radar plot showing the mean values of each of the four connectivity indices for the four types of undirected graphs analyzed. Index I1: transport efficiency. Index I2: mean node degree. Index I3: mean node PageRank. Index I4: maximum node PageRank. The graph obtained with the calibrated R-SWMS model lays closer to the experimental graph than the graphs obtained with the benchmark network algorithms (fan tree and Steiner tree).

## Appendix: RSWMS model verification against network performance indices

We transformed RSAs obtained numerically as undirected graphs for 27 cases, in which $W_g \in [0.1, 0.2]$, $W_r \in [0.35, 0.45]$ and $\delta \in [25^\circ , 35^\circ ]$. The seed (the initial root segment), the endpoints and the branching points are the nodes of the graph. The portions of root segments in between are the edges of the graph. In the simulations, the average axis growth rate, the average secondary branch root rate, the average branching angle and the average branch spacing were the ones measured experimentally. The tip delay time was calibrated in the “Numerical modeling and discussion” section (2.1 days). The water fluid flow parameters were the same as those described in Table 2. We then generated the Steiner trees and the fan trees that have the same seed and endpoints as the undirected graphs obtained numerically. The fan tree is obtained by drawing a straight line between the seed and each of the endpoints, i.e., there is no branching node. The Steiner tree is the minimum spanning tree that can be obtained when the introduction of extra branching points (Steiner points) is allowed. Of course, the Steiner trees do not have the same branching nodes as the undirected graphs obtained with the R-SWMS model. We call the Steiner tree and the fan tree networks “benchmark networks”. Figure 11 shows the graph obtained from the experiment as well as one of the graphs obtained numerically with the R-SWMS model, along with the two corresponding benchmark networks.

We quantify graph topology and connectivity with indices that are independent of their length scale, so that a comparison between the experimental and numerical networks is possible. The following network indices are defined:

1. Transport efficiency: the sum of the travel distances from the seed to each node, normalized by the total network length (note that since the fan tree has no branching node, its transport efficiency index is one, while all other networks exhibit a higher transport efficiency index).
2. Mean graph node degree: the degree of a node is defined as the number of edges connected to it. Therefore, the mean node degree of the graph is an index of its branching patterns: the branching density increases with the mean node degree.
3. Mean graph node PageRank: the PageRank betweenness of a node is defined as a the probability that the given node will be part of the shortest path between any other pair of nodes in the network. Thus, the mean node PageRank of the network is an index describing the connectivity distribution of the nodes in a network. Higher values correspond to more connected, balanced networks while lower values correspond to graphs where just a few nodes connect all the other nodes.
4. Maximum node PageRank: the maximum PageRank betweenness is a measure of the connectivity balance of the graph; higher values are characteristic of highly centralized networks.

Statistical boxplots that illustrate the variability of the indices are shown in Fig. 12. All algorithms display very low variability in terms of mean node degree and in terms of node importance (mean and maximum node PageRank). This observation suggests that the optimization criteria used in each algorithm consistently yields the same network topology. We also note that the RSA graphs obtained from the R-SWMS model and from the experiment exhibit more branching and more even distribution of node importance when compared to the benchmark networks, and a better transport efficiency than the Steiner tree, close to that of the fan tree. Moreover, the coordination indices of the RSA graph obtained experimentally are closer to those of the RSA graph obtained with the R-SWMS model than with any of the benchmark networks, which confirms that the R-SWMS model was properly calibrated. The radar plot shown in Fig. 13 summarizes the network index analyses.
