# Supplementary material for: Modeling root system growth around obstacles
Source: Sci Rep. 2020 Sep 28;10:15868. doi: 10.1038/s41598-020-72557-8 (PMC7522252; doi:10.1038/s41598-020-72557-8)
Supplement: Supplementary file 1 — Supplementary material 1 [file 41598_2020_72557_MOESM1_ESM.docx]

## *Scientific Reports* Supporting Figures

Article title: Modeling root system growth around obstacles

Authors: Wencheng Jin, Jayde Aufrecht, Fernando Patino-Ramirez, Heidy Cabral, Chloe Arson, Scott Retterer

Corresponding author:

Wencheng Jin

Energy and Environmental Science & Technology Directorate, Idaho National Laboratory, ID

[wencheng.jin@inl.gov](mailto:wencheng.jin@inl.gov)

**Fig. S1** Photographs of root systems growing around rectangular obstacles placed at different inclination angles $\eta$, taken at different growth times $t$.

**Fig. S2** Extracted growth rate of root axes after the major axis has hit a rigid obstacle, for various obstacle inclination angles $\eta$. Results are shown for three replicate experiments for each orientation angle. The horizontal axis indicates the time elapsed after the major axis has hit the obstacle. In order to better highlight the influence of obstacles on the growth rate of the major axis (the first root axis that emerges from the seed), we shifted the growth time shown on the x-axis such that a zero coordinate corresponds to the time when the major axis contact the obstacle. The black dots represent the evolution of the growth rate of the major axis, while the red dots represent the evolution of the average growth rate of the secondary axes. We calculated the average growth rate of all the secondary axes (note that the experiments were stopped before the occurrence of second-order (lateral) roots). The comparison between the black and the red dots provides an indication of how the nutrients are distributed between the axes after the major axis has hit the obstacle.

**Fig. S3** Calibration of the tip delay time. The total length is defined as the total root length for all axes and laterals. The least square root of the error between the total branch length evolution obtained numerically and that obtained experimentally is minimal for a tip delay time of 2.1 days.

**Fig. S4** Parametric study on the influence of the geotropism weight $W_{g}$ on the RSA. Note $W_{r}=0.5$, $\delta={50}^{\circ},$ are kept constant. From the comparison between experiments and predictions, the range of geotropism weight can be narrowed down as $W_{g}\in[0.1,0.2]$.

**Fig. S5** Parametric study on the influence of the penetration resistance gradient weight $W_{r}$ on the RSA. Note $W_{g}=0.2$, $\delta={50}^{\circ}$ are kept constant. From the comparison between experiments and predictions, the range of penetration resistance gradient weight can be narrowed down as $W_{r}\in[0.35,0.45]$.

**Fig. S6** Parametric study on the influence of the random deviation angle $\delta$ on the RSA. Note $W_{g}=0.2$, $W_{r}=0.4$ are kept constant. From the comparison between experiments and predictions, the range of random deviation angle can be narrowed down as $\delta\in[{25}^{\circ},{35}^{\circ}]$.
